# A Special Section of Papers From: ISMB BioLink 2004: “Linking Literature, Information and Knowledge for Biology”

**DOI:** 10.1002/cfg.458

**Published:** 2005


					Comparative and Functional Genomics
A Special section of papers from
ISMB BioLink 2004:
``Linking Literature, Information and
Knowledge for Biology''
A Special Interest Group (SIG) Workshop of the
12th
Intelligent Systems for Molecular Biology (ISMB)
&
3rd
European Conference on Computational Biology (ECCB)
Held at the Moat House Hotel, Glasgow, 29th
July 2004
With editorial assistance from
Lynette Hirschman
Christian Blaschke
Alfonso Valencia
Program Committee
Christian Blaschke, CNB Madrid, Spain
Luc Deshaspe, PharmaDM, Belgium
Robert Gaizauskas, University of Sheffield, UK
William Hersh, Oregon Health & Science University, USA
Lynette Hirschman, MITRE, USA
Alfonso Valencia, CNB Madrid, Spain
Karin Verspoor, Los Alamos National Laboratory, USA
Alexander Yeh, MITRE, USA

				

